# Bowel cleansing efficacy of 1 L NER1006 *versus* macrogol and 3 L polyethylene glycol using split‐dose administration

**DOI:** 10.1002/jgh3.12816

**Published:** 2022-11-05

**Authors:** Priya Sinh, Katherine Dunn, Sneha John

**Affiliations:** ^1^ Department of Pharmacy Gold Coast University Hospital Gold Coast Queensland Australia; ^2^ Department of Gastroenterology and Digestive Health Gold Coast University Hospital Gold Coast Queensland Australia

**Keywords:** bowel cancer screening, bowel preparation, colonoscopy, colorectal cancer, macrogol, macrogol and polyethylene glycol, NER1006, polyethylene glycol, polyethylene glycol plus ascorbate, polyp detection rate

## Abstract

**Background and Aim:**

Colonoscopies are an important diagnostic technique in the detection of colorectal cancer and colonic disease. Adequate examination is dependent on the degree of mucosal visibility, with poor cleansing impeding the detection of neoplasms. These patients require shorter colonoscopy surveillance intervals, longer hospital stays, and increased healthcare costs—rendering a screening colonoscopy cost‐ineffective. In Australia and the Gold Coast Hospital and Health Service (GCHHS), macrogol and 3 L of polyethylene glycol are the preferred regimen given its safety profile and efficacy. Yet, little is known locally about the use of the new low‐volume bowel preparation NER1006 (Plenvu) given its recent registration with the Therapeutic Goods of Australia (TGA). The primary outcome assessed the bowel cleansing efficacy of NER1006 compared with 7 days of macrogol and 3 L of polyethylene glycol using the Boston Bowel Preparation Scale (BBPS), while also assessing the influence of notable patient characteristics such as age, gender, body mass index (BMI), and the patients Charlson comorbidity index (CCI). Secondary outcomes assessed the polyp detection rate and procedural factors including cecal intubation, scope withdrawal time, and rebooking rates.

**Methods:**

Data from all patients who underwent an outpatient colonoscopy procedure at GCHHS between 1 July 2020 and 30 September 2020 were analyzed. Patients were aged 50–74 years of age and were referred for a screening colonoscopy due to a positive fecal occult blood test (FOBT) result from the National Bowel Cancer Screening Program.

**Results:**

Of the 238 patients who met the inclusion criteria, 108 patients received NER1006 and 130 patients received macrogol and 3 L polyethylene glycol. NER1006 achieved superior overall (*P* < 0.001) and right‐sided colon cleansing (*P* = 0.016). There was an even distribution of males and females (*P* = 0.118), the mean age of both cohorts was <65 years of age. The macrogol and 3 L polyethylene glycol group had a statistically higher BMI (*P* < 0.001) and CCI (*P* < 0.001). Cecal intubation success was achieved in both cohorts (≥95%) and scope withdrawal time was ≥6 min, polyp detection was non‐superior (*P* = 0.824), but superior in NER1006 when BBPS ≥6 (*P* = 0.002). Rebooking rates were significantly lower in the NER1006 group (*P* = 0.013).

**Conclusion:**

This study demonstrated that NER1006 was superior in terms of overall and right‐sided bowel cleansing as a primary endpoint. Patient factors demonstrated to be independent predictors of inadequate bowel preparation. Future studies should aim to explore the safety and tolerability of NER1006 while also assessing the bowel cleansing effectiveness in patients with a high BMI and comorbidity index.

## Graphical abstract

In Australia, high‐volume polyethylene glycol based bowel preparations is our gold standard to achieve acceptable levels of colon cleansing for a colonoscopy. Yet, the rebooking rates and subsequent costs to the health service warranted further investigation to determine whether other bowel preparation options were more effective, while maintaining a safety profile. The results from our study demonstrated that NER1006, a novel 1 L low‐volume polyethylene‐based bowel preparation produced significant results compared with its counterpart of macrogol and 3 L polyethylene glycol (*P* < 0.001). The results from this study will provide more confidence when prescribing NER1006, especially in patients who are intolerant to high‐volume preparations.

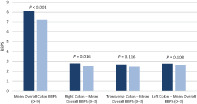

## Introduction

Colonoscopies are an important diagnostic method in the detection of colorectal cancer (CRC) and colonic disease.^1^ In 2016, CRC was the third most common diagnosed cancer in Australia, with a 70% 5‐year survival rate.^2^ The high mortality rate associated with this type of cancer reinforces the importance of diagnostic accuracy in the early detection of neoplasms, which can be compromised when colonic preparation quality is inadequate.^3^ CRC contributes a significant cost burden to the healthcare system and the individual, with an estimated 16 000 new diagnosis made in Australia in 2020 alone.^2^

Adequate bowel cleansing is crucial in the success of colonoscopy. However, inadequate bowel cleansing is observed in approximately 25% of all colonoscopies.^3^ The implications of insufficient colon cleansing include failed cecal intubation and decreased adenoma detection, prolonged procedural times, and shorter surveillance intervals—rendering a screening colonoscopy cost‐ineffective.^4^ Hence, it is imperative to improve the quality of bowel preparation.

In Australia, there are numerous bowel cleansing solutions available. Polyethylene glycol (PEG) based bowel preparations are recognized as first‐line due to their superior safety profile over other solutions containing sodium picosulfate and sodium phosphate.^5^ These high‐volume, osmotically balanced PEG solutions impair the intestinal absorption of water and sodium by maintaining isosmosis in the bowel lumen.^6^ Previous studies have demonstrated that PEG‐based preparations have fewer contraindications and electrolyte disturbances. However, they generally require dilution with a high volume of water, which can often cause poor patient tolerability and experience.

NER1006 (Plenvu) is the first low‐volume 1 L‐PEG based bowel preparation to show superior high‐quality segmental cleansing of the colon compared with other preparations on the market.^7^ NER1006 contains an osmotically active ascorbic acid with essential electrolytes to prevent dehydration and is a taste optimised formulation consisting of two sachets. The first sachet consists of macrogol only and the second sachet consists of macrogol and high‐dose ascorbate (55.65 g).^8^ The contrast in flavors aims to reduce flavor fatigue and improve the overall tolerability and experience for patients.^9^

There are many independent factors that play a significant role in bowel preparation adequacy.^5^ Typically age (≥65 years), male gender, body mass index (BMI) greater than 30, diabetes, and smoking are factors that have been associated with poor bowel cleansing in the literature.^5^

To reduce the incidence of suboptimal bowel preparation, research has focused on the timing of dosing in relation to a colonoscopy, with numerous studies showing that a split‐dose regime is superior to day before dosing.^10^ A split‐dose regime is where the patient takes a portion of the preparation the evening prior and another portion on the day of the colonoscopy to improve bowel preparation quality, patient compliance, and tolerability. Yet, there is still limited research on how patient risk factors interact with split‐dose regimes.

Inadequate bowel preparation is also a strong predictor of cecal intubation failure and poor patient experience.^8^ Cecal intubation time is often used as a surrogate to estimate the difficulty of colonoscopy, with shorter cecal intubation times being correlated with the increased detection of polyps.^11^ Patient factors such as age, BMI, and prior abdominal surgery have been associated with increased cecal intubation times, thus prolonging procedural time. Another study also demonstrated that the mean number of adenomas detected per patient decreased as cecal intubation time increased by 7–11% per time quartile.^12^ Therefore, it is also important to identify patient risk factors early on, which can allow us to tailor protocols to achieve optimal preparation efficacy. Yet, defining the clinical predictors of inadequate bowel cleansing remains an ambitious topic.

Globally, there are no studies that examine the bowel cleansing efficacy of high‐volume 3 L‐PEG based solution (GlycoPrep‐C) compared with NER1006 (Plenvu). Given that PEG‐based preparations are first‐line in Australia, this warrants examination as NER1006 is relatively new to the Australian market and has produced promising results in overseas studies. At GCHHS, the utiliation of a split‐dose regimen of 3 L‐PEG with the addition of macrogol (Movicol) for 7 days prior is our current gold standard.

## Methods

### Study design

**Figure 1:**
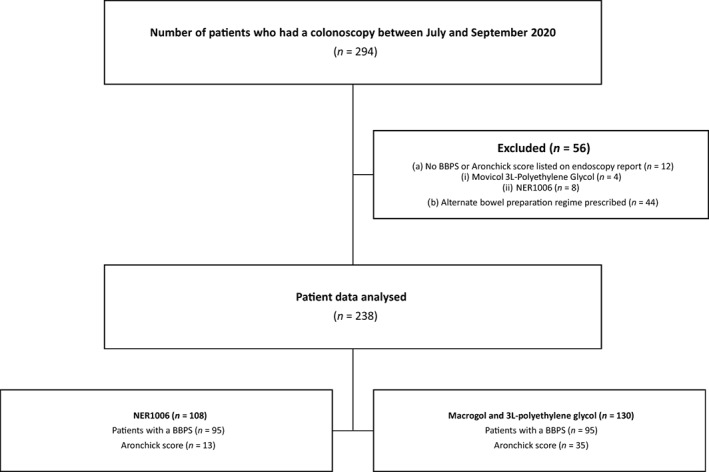
Exclusion flowchart. BBPS, Boston Bowel Preparation Scale.

This study was a retrospective, single‐center analysis conducted at a tertiary center at the GCHHS. The data of 294 colonoscopies between 1 July to 30 September 2020 were analyzed (Fig. 1). Procedural staff were only made aware of the prescribed bowel preparation if the patient had achieved a fair or poor bowel cleansing result (BBPS <6). Macrogol (Movicol) and 3 L‐PEG (GlycoPrep‐C) and NER1006 (Plenvu) were the two regimes assessed in this study.

### Study population

Study patients included men and women aged 50–74 years of age who had received a positive fecal occult blood test from the test kit provided by the Australian National Bowel Cancer Screening Program (NBCSP). Clinical nurses allocated patients into an AM (7:30–12:00 h) or a PM (after 12:00 h) list. Diabetic patients and social admissions took preference for a morning admission. A split‐dose regime was utilized in both groups.

### AM procedure (07:30–12:00 h)

*NER1006*: Patients were instructed to self‐administer the first dose at 18:00 the night before and second dose at 04:00 the day of the procedure. Patients were nil by mouth at 05:30.

*Macrogol and 3 L‐PEG*: Macrogol was prescribed as 1–2 sachets once a day, commencing 7 days prior to the procedure. The first two sachets of 1 L‐PEG were prescribed at 16:00 and 18:00 the day before and the third dose at 04:00 on the day of the colonoscopy. Patients were nil by mouth at 05:30.

### PM procedure (12:00 h onward)

*NER1006*: Patients were instructed to self‐administer the first dose at 20:00 the night before and second dose at 08:00 the day of the procedure. Patients were nil by mouth at 11:00.

*Macrogol and 3 L‐PEG*: Macrogol was prescribed as 1–2 sachets once a day, commencing 7 days prior to the procedure. The first dose of 1 L‐PEG was prescribed at 17:00 the day before. The second and third sachets at 07:00 and 09:00 respectively on the day of the procedure. Patients were nil by mouth at 11:00.

There was a >2 h window between finishing the bowel preparation and the procedure taking place. Patients were instructed to be nil by mouth for at least 2 hours prior to the procedure. For all patients, the administration of the bowel preparation took place at home as an outpatient.

Written and verbal instructions regarding dietary modifications, timing of bowel preparation consumption, and with‐holding medications were explained at either a clinic or telephone review prior to their procedure to ensure uniformity. Three days before the procedure, patients were instructed to commence a low fiber diet. The last meal was consumed by 13:00 the day prior to the procedure. After this, patients were only able to consume clear fluids. Patients were provided with an information leaflet with strict instructions requiring dietary modifications and timing of bowel preparation consumption. Patients who obtained their bowel preparation from the hospital pharmacy received in‐person counseling, additional to their telephone or in‐person clinic review, which also thoroughly explained bowel preparation instructions. Exactly 69.4% of patients obtained NER1006 and 86% obtained their macrogol and 3 L‐PEG preparations from the GCHHS hospital pharmacy. All other patients obtained their medication from their local community pharmacy.

### Data collection

Patient characteristics collected include age, gender, BMI, smoking status, Charlson comorbidity index (CCI) score, and procedural factors. The CCI predicts the 10‐year mortality for a patient with a range of comorbid conditions and takes into consideration important factors such as, but not limited to: age, diabetes mellitus, chronic obstructive pulmonary disease (COPD), and cancer.^13^ Procedural factors included bowel cleansing effectiveness using the Boston Bowel Preparation Scale (BBPS), procedural factors included cecal intubation success, scope withdrawal time, and polyp detection rate (PDR).

The primary outcome assessed the bowel cleansing efficacy of NER1006 compared with 7 days of macrogol and 3 L‐PEG using the Boston Bowel Preparation Scale (BBPS) as a standardized measure; while also assessing the influence of notable patient characteristics such as age, gender, BMI, and their CCI. The BBPS grades the quality of bowel preparation in the right, transverse, and left colon.^14^ Each segment is scored from 0 to 3 with a score of 9 being a total of all three segments combined. A score of 0 refers to “*an unprepared colon segment with mucosa not seen due to solid stool that cannot be cleared*” and a score of 3 denotes that the “*entire mucosa of colon segment seen well, with no residual staining, small fragments of stool or opaque liquid*.”^14^ If the cleanliness score on the colonoscopy report was per the Aronchick scale (AS) of excellent, good, fair, poor, or inadequate, this was then converted to a BBPS equivalent through the following criteria. An excellent and good result was denoted as a BBPS ≥6, “*clear liquid covering up to 25% of mucosa, but ≥90% of mucosa seen*.” Whereas a fair, poor, or inadequate result was denoted as a BBPS <6 whereby “*semi‐solid stool could not be suctioned and <90% of mucosa seen*.”^15^

Secondary outcomes assessed the PDR and procedural factors including cecal intubation and scope withdrawal time, while also assessing the impact on bowel preparation quality. Cecal intubation success is defined as a successful advancement of the colonoscope to the cecum, identified by the appendiceal orifice and ileocecal valve. Withdrawal time is defined as the time taken from cecal intubation to withdrawal of the colonoscope from the anus and includes intervention time (e.g. polypectomy or biopsy) in this study.^16^

### Data analysis

Statistical analysis was performed using the IBM Statistical Package for the Social Sciences (SPSS) for MAC, version 28.0.1.1 (14).

Patient characteristics were reported using simple statistics such as *n* (number of patients) and % (percentage of patients) and mean (SD). Two‐sided *t*‐tests were used to compare the means of the variables between two groups, while the Pearson chi test and Fishers exact test were used to compare categorical variables. These values were evaluated using a *P* < 0.05.

Multivariate logistic regression (MANOVA) analysis was performed on the data set to identify the relationship between three or more variables and was used to determine whether independent patient characteristics or procedural factors influenced bowel preparation quality. This was using a Bonferroni adjusted level of 0.025, with *P* values less than 0.025 indicating statistical significance.

## Results

The eligibility of 294 patients was evaluated in this study as seen in Figure 1. These patients were recruited consecutively from a list who were undergoing a screening colonoscopy. Colonoscopies were performed by both gastroenterologists and nurse practitioner endoscopists (NPE).

### Patient characteristics

Overall, patient characteristics were well balanced between the two groups (Table 1). The results demonstrated that there was an even distribution between both genders. There were 46.29% (*n* = 50) and 56.92% (*n* = 74) of males in each group respectively (*P* = 0.118). Patients who were ≥65 years or older was 24.07% (*n* = 26) and 41.53% (*n* = 54) in the macrogol and 3 L polyethylene glycol group. Patients who were less than 65 years of age was 75.92% (*n* = 82) and 58.46% (*n* = 76) respectively. The mean differences in age were statistically significant between the two groups (*P* = 0.003), favoring a cohort less than 65 years of age. A difference was noticed in the mean BMI between the two groups (*P* < 0.001), with notable positioning problems experienced by proceduralists in the macrogol and 3 L polyethylene glycol cohort when a BMI exceeded 40 kg/m^2^. Smoking status was denoted as an active smoking status at the time of the procedure. Exactly 15.38% (*n* = 20) of patients in the NER1006 were smokers and 19.44% (*n* = 21) (*P* = 0.731) respectively. The CCI was used to calculate the comorbidity burden of patients in both groups 1.78 ± 0.998 *versus* 2.43 ± 1.44 (*P* < 0.001). There was a higher proportion in the mean CCI in the macrogol and 3 L polyethylene glycol group.

**Table 1:** Patient characteristics

Characteristic	NER1006 (*n* = 108)	Macrogol and 3 L polyethylene glycol (*n* = 130)	*P* value
Sex, *n* (%)			
Male	50 (46.29)	74 (56.92)	*P* = 0.118^†^
Female	58 (53.70)	56 (43.07)	
Age, *n* (%)			
≥65 years	26 (24.07)	54 (41.53)	
<65 years	82 (75.92)	76 (58.46)	
Mean, SD	59.32 ± 7.04	62.15 ± 7.28	*P* = 0.003^‡^
BMI (kg/m^2^)			
<18.5	4 (3.70)	0	
18.5–24.9	39 (36.11)	41 (31.53)	
25–29.9	39 (36.11)	40 (30.76)	
30–34.9	23 (21.29)	29 (22.30)	
35–39.9	3 (2.77)	10 (7.69)	
40 or more	0	10 (7.69)	
Mean, SD	26.32 ± 4.76	28.99 ± 5.71	*P* < 0.001^‡^
Smoking status			
*n* (%)	20 (15.38)	21 (19.44)	*P* = 0.731^§^
Male	16	15	
Female	4	6	
Charlson comorbidity index, *n* (%)			
0–2 points (98–90%), 10 year survival	82 (75.92)	75 (57.69)	
3–4 points (77–53%), 10 year survival	24 (22.22)	44 (33.84)	
≥5 points (21–0%), 10 year survival	2 (1.85)	11 (8.46)	
Mean, SD	1.78 ± 0.998	2.43 ± 1.44	*P* < 0.001^‡^

^†^ *P* value calculated from Fisher's exact test for the difference between treatment groups.

^‡^ *P* value calculated using two‐sample *t*‐test.

^§^ *P* value calculated from Fisher's exact test for the difference between treatment groups.

*n* = number of patients; % = percentage of patients. Smoking status = active smoking status at time of procedure. Charlson comorbidity index = defined as 10 year survival rate.

BMI, body mass index (kg/m^2^).

### Bowel cleansing effectiveness

**Figure 2:**
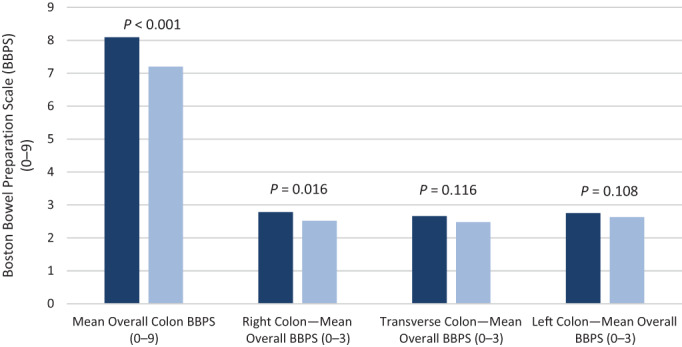
Bowel cleansing effectiveness according to Boston Bowel Preparation Scale (BBPS) for overall, right, transverse, and left colon. (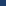), NER1006; (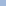), macrogol and 3 L polyethylene glycol.

NER1006 achieved superiority compared with macrogol and 3 L polyethylene glycol for successful overall and right‐sided colon cleansing (BBPS: 8.09 *vs* 7.20) (*P* < 0.001) (Table 2). Patients who achieved an overall BBPS ≥6 in the NER1006 group was 91.66% (*n* = 99) and 83.07% (*n* = 108) in the macrogol and 3 L polyethylene glycol group (*P* < 0.001). Bowel cleansing in the right colon segment also achieved a significant result (BBPS: 2.78 *vs* 2.52) (*P* = 0.016), while left colon cleansing was non‐inferior (BBPS: 2.75 *vs* 2.63) (*P* = 0.108). Cleansing in the transverse colon was also non‐inferior (2.66 *vs* 2.48) (*P* = 0.116), illustrated in Figure 2.

**Table 2:** Bowel cleansing efficacy and procedural factors

*n* (%)	NER1006 (*n* = 108)	Macrogol and 3 L polyethylene glycol (*n* = 130)	*P* value
Bowel cleansing efficacy mean (BBPS)—Median (IQR)	8.09 (8–9)	7.20 (7–9)	*P* < 0.001^†^
Patients with BBPS ≥6	99 (91.66)	108 (83.07)	*P* < 0.001^‡^
Patients with BBPS <6	9 (8.33)	22 (16.92)	
Right colon—Mean overall BBPS (0–3)	2.78	2.52	*P* = 0.016^‡^
Transverse colon—Mean overall BBPS (0–3)	2.66	2.48	*P* = 0.116^‡^
Left colon—Mean overall BBPS (0–3)	2.75	2.63	*P* = 0.108^‡^
Gender			
Males with BBPS ≥6	49 (98)	47 (85.13)	*P* = 0.11
Females with BBPS ≥6	58 (100)	36 (87.5)	
Age			
Patients ≥65 and BBPS ≥6	23 (88.46)	47 (87.03)	*P* = 0.001^§^
Patients <65 and BBPS ≥6	76 (92.68)	61 (80.26)	
Procedural factors			
AM procedural list	72 (66.6)	78 (60)	*P* = 0.345^¶^
PM procedural list	36 (33.3)	52 (40)	
AM procedural list and PDR when BBPS ≥6	65 (90.27)	66 (84.61)	*P* = 0.628^‡^
PM procedural list and PDR when BBPS ≥6	34 (94.44)	42 (80.76)	
Cecal intubation success	107 (99)	124 (95.38)	*P* = 0.131^‡^
Scope withdrawal time—Median (min) (IQR)^††^	10.61 (5.72)	13.69 (10.45)	*P* = 0.486^‡^
Withdrawal time—BMI ≥ 30 kg/m^2^ (min) (IQR)	13.23 (10.25)		*P* = 0.642^‡^
Withdrawal time—BMI < 30 kg/m^2^ (min) (IQR)	11.59 (7.45)		
Withdrawal time—≥ 65 years of age (min) (IQR)	13.95 (9.55)		*P* = 0.441^‡^
Withdrawal time—< 65 years of age (min) (IQR)	11.18 (7.033)		
Withdrawal time—CCI ≥ 4 (min) (IQR)	15 (8.16)		*P* = 0.489^‡^
Withdrawal time—CCI < 4 (min) (IQR)	11.30 (10.55)		
Withdrawal time—Male (min) (IQR)	13.23 (8.55)		*P* = 0.329^‡^
Withdrawal time—Females (min) (IQR)	11.03 (7.35)		
Polyp Detection Rate (PDR)	81 (75)	101 (77.69)	*P* = 0.824^¶^
PDR when BBPS ≥6	80 (80.80)	90 (83.33)	*P* = 0.002^‡^
PDR when BBPS <6	1 (11.11)	11 (50)	
Patients rebooked within 12 months	3 (2.77)	16 (12.30)	*P* = 0.013^¶^

^†^ *P* value calculated using two‐sample *t*‐test.

^‡^ *P* value calculated using Pearson chi‐square test.

^§^ *P* value calculated using multivariate analysis.

^¶^ *P* value calculated using Fisher's exact test.

^††^ IQR range (9.29–17.90 min).

*n* = number of patients; % = percentage of patients. BBPS = total Boston Bowel Preparation Score from 0 to 9. NB = each colon segment is evaluated from 0 to 3. Right colon includes = right colon, sigmoid, and rectum. Cecal Intubation Success = ability of the endoscopist to reach the caecum. Scope Withdrawal Time = inc. intervention time. Time to withdraw the scope from the cecum to the anus. CCI = Charlson comorbidity index whereby a score of 4 indicates a 53%, 10‐year survival rate. Rebooked = number of patients rebooked due to poor bowel cleansing (BBPS < 6).

IQR, interquartile range; PDR, polyp detection rate.

Upon comparison of patients aged above and below 65 with a BBPS ≥6, patients who were <65 years of age had statistically superior bowel cleansing outcomes (*P* < 0.001). Patients who were ≥65 years and who achieved a BBPS ≥6 was 88.46% (*n* = 23) and 87.03% (*n* = 47) respectively. Those <65 years were 92.68% (*n* = 76) and 47.36% (*n* = 61) (*P* < 0.001), demonstrating that a greater proportion of patients who achieved a BBPS ≥6 was <65 years of age.

### Procedural factors

**Figure 3:**
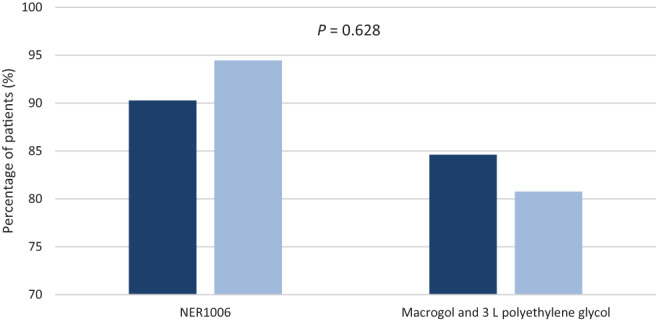
Patient distribution between AM and PM procedure list when Boston Bowel Preparation Scale (BBPS) ≥6. (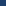), AM procedural list—BBPS >6; (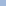), PM procedural list—BBPS >6.

There was a similar proportion of patients allocated to the AM list 66.6% (*n* = 72) and 60% (*n* = 78) compared with the PM list, which was 33.3% (*n* = 36) and 40% (*n* = 52), (*P* = 0.345) respectively. Both procedural lists saw a similar proportion of patients who achieved an adequate bowel cleansing result of BBPS ≥6 (*P* = 0.628), which is illustrated in Figure 3.

Cecal intubation was achieved in 99% (*n* = 107) of patients in the NER1006 group and 95.38% (*n* = 124) in the macrogol and 3 L polyethylene glycol group (*P* = 0.131), demonstrating non‐inferiority. Scope withdrawal time between the two groups were also similar 10.61 min (interquartile range [IQR] 5.72) and 13.69 min (IQR 10.45) (*P* = 0.486). Scope withdrawal time including intervention time in males was 13.23 min (IQR 8.55) and females 11.03 min (IQR 7.35) (*P* = 0.329). The median scope withdrawal time of patients ≥65 years of age was 13.95 min (IQR 9.55) and <65 years of age 11.18 min (IQR 7.033) (*P* = 0.441). Patients with a BMI ≥30 kg/m^2^ had a median withdrawal time of 13.23 min (IQR 10.25) and a BMI <30 was 11.59 min (IQR 7.45) (*P* = 0.642). Patients with a CCI ≥4 indicating a moderate comorbidity burden and a 53%, 10‐year survival rate had a median withdrawal time of 15 min (IQR 8.16) and CCI <4, 11.30 min (IQR 10.55) (*P* = 0.489). Furthermore, multivariate analysis testing reaffirmed this as shown in Table 3, the MANOVA test showed significance when comparing scope withdrawal time and polyp detection. Demonstrating that there is a correlation between these two factors (*P* = 0.004). There was a statistically significant pull toward patients ≥65 with a higher CCI ≥4 (*P* < 0.001). Male patients also displayed a higher BMI ≥30 kg/m^2^ (*P* = 0.096) and CCI ≥4 (*P* = 0.433).

**Table 3:** Multivariate analysis of procedural factors

Tests of between‐subject effects		
Source	Dependent variable	Significance if *P* < 0.025
Scope withdrawal time	Gender (male *vs* female)	*P* = 0.129
	Age (≥65 *vs* <65)	*P* = 0.143
	BMI (≥30 kg/m^2^ *vs* <30 kg/m^2^)	*P* = 0.610
	Charlson comorbidity index score (≥4 *vs* <4)	*P* = 0.156
	Total resected polyps	*P* = 0.004
Age	Charlson comorbidity index	*P* < 0.001
Gender	BMI	*P* = 0.096
	Charlson comorbidity index	*P* = 0.433

BMI, body mass index.

**Figure 4:**
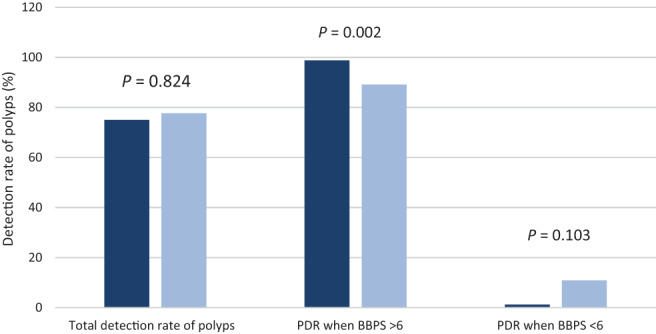
Overall detection rate of polyps (PDR) between both groups and detection rate when Boston Bowel Preparation Scale (BBPS) ≥6 and <6. (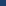), NER1006; (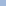), macrogol and 3 L polyethylene glycol.

The detection rate of polyps in the NER1006 group was 75% (*n* = 81) and 77.69% (*n* = 101) in the macrogol and 3 L polyethylene glycol group (*P* = 0.824). In patients who had a BBPS ≥6, 98.76% patients had polyps identified, compared with 89.10% of patients respectively. When inadequate bowel cleansing was achieved, BBPS < 6, 1.23% patients had polyps identified compared with 10.89% of patients. Patients who achieved a BBPS ≥6 had a significant PDR (*P* = 0.002), which is illustrated in Figure 4. Rebooking rates were statistically significant between the groups. 2.77% (*n* = 3) patients were rebooked in the NER1006 group due to poor preparation and 12.30% (*n* = 16) in the macrogol and 3 L polyethylene glycol group (*P* = 0.013).

## Discussion

Effective bowel preparation is crucial for a quality colonoscopy procedure. It allows for optimal visualization and appropriate diagnosis. While preparation compliance is one of the most important factors in determining bowel preparation adequacy, patient characteristics and procedural factors also play a role.

The primary outcome of this study was to assess the bowel cleansing effectiveness of NER1006 compared with 7 days of macrogol and 3 L polyethylene glycol, which is the current gold standard at GCHHS. Overall, bowel cleansing superiority was demonstrated in NER1006 *versus* macrogol and 3 L polyethylene glycol on mean overall BBPS (BBPS mean: 8.09 *vs* 7.20) (*P* < 0.001). NER1006 also demonstrated statistically significant cleansing in the right colon and favorable results in both the transverse and left colon. Right‐sided colon cleansing also produced statistically favorable results (*P* = 0.016), which is notable as high‐risk sessile serrated polyps are located in this area and account for a higher mortality compared with left‐sided lesions.^17^ Prior studies have suggested that the enhanced osmotic activity of NER1006 enables more effective right‐sided cleansing.^9^ Consistent with the MORA study, the outcomes of our study produced similarly favorable results whose outcomes showed superiority of NER1006 compared with 2 L polyethylene glycol (MoviPrep) (*P* < 0.001) based on mean overall and right colon BBPS. This supports the use of NER1006 as a suitable option, which aligns with current prescribing preferences in Australia. The benchmark for adequate bowel preparation as set by the Quality Committee of the European Society of Gastrointestinal Endoscopy (ESGE) stipulates that for screening or surveillance scopes, “*a 90% minimum standard for adequate bowel preparation*.”^6^ In our study, 13.02% percent of our overall study population had inadequate bowel preparation, defined as BBPS <6 (inadequate, fair or poor), which falls outside these parameters and has become a surrogate for further sources of improvement within our department.

Patient characteristics such as age, gender, BMI, and the CCI were independent factors examined to assess bowel cleansing adequacy. Several studies have evaluated age ≥65 as a predictor of poor bowel preparation. Increased age is known to be associated with slower colonic transit due to degeneration of the autonomic nervous system.^5^ Patients with multiple comorbidities exhibit polypharmacy and tend to be immobile, resulting in constipation and poor bowel preparation. The male gender and a BMI ≥30 kg/m^2^ are also known independent attributing factors.^12^

Multivariate analysis conducted on the data set demonstrated that patients ≥65 years of age had a higher CCI which was statistically significant (*P* < 0.001). Males had a higher BMI (*P* = 0.096) and CCI (*P* = 0.433), indicating that the macrogol and 3 L polyethylene glycol group had patients with a higher comorbidity burden, which may have resulted in inadequate preparation. Given that this study was conducted in 2020 when NER1006 was very new to GCHHS, cautious prescribing may have been employed and more conservative measures may have been executed to ensure a clean bowel prior to the procedure. In the future, lenient prescribing in patients with a high BMI or CCI would accurately assess NER1006's effectiveness in this cohort who are susceptible to higher procedural complications and adverse effects.

Secondary outcomes assessed the PDR and procedural factors including cecal intubation and scope withdrawal time, while also assessing the impact on bowel preparation quality.

In our study, PDRs were noted to be similar in both arms with no significant difference 75% (*n* = 75) *versus* 77.69 (*n* = 101), (*P* = 0.824) (Table 2). Yet, further analysis shows that PDR was higher when BBPS ≥6 (*P* = 0.002). These results show that the detection of polyps is affected in patients who achieve a BBPS <6, as colonic preparation quality is compromised, with our results also aligning with those from the retrospective audit conducted by the ASGE.^18^ Both groups saw a similar number of patients booked into the AM and PM procedural lists, with more patients receiving an AM procedure (66.6% *vs* 33.3%) *versus* PM procedure (60% and 40%) respectively (*P* = 0.345). NER1006 had a higher PDR in the PM list when BBPS ≥6 (94.44%) and macrogol and 3 L polyethylene glycol in the AM list (84.61%), this was not significant (*P* = 0.628). European guidelines have defined an overall aim of 40% PDR in all colonoscopies.^19^, ^20^, ^21^ Both groups exceeded this 40% detection rate, which could be attributed to both preparations being administered as a split‐dose regimen whose outcomes can be reaffirmed by a study done in 2015 by Radaelli *et al*. whereby split regimes resulted in a higher detection of neoplastic lesions irrespective of time of day.^22^ Furthermore, the osmotically active ascorbic acid in the second sachet of NER1006 could be responsible for a higher PDR in the PM list.

Cecal intubation success and scope withdrawal times can indicate the difficulty of a colonoscopy. Lower cecal intubation rates correlate with higher rates of cancer.^17^ Our study achieved 99% cecal intubation success in the NER1006 cohort and 95.38% in the macrogol and 3 L polyethylene glycol cohort. These values align with the performance indicators set out by the National Bowel Cancer Screening Program Quality Working Group (NBCSPQWG), with 90% for general patients and 95% for patients undergoing screening colonoscopies.^17^ Prolonged withdrawal times in the literature have demonstrated an increased risk of adverse events and potential complications from anesthesia.^23^ This study showed that those patients who were male, a BMI ≥30 kg/m^2^, ≥65 years of age, and a CCI ≥4 all had longer median scope withdrawal times, although were not statistically significant. It is therefore important to understand the presence of risk factors that can indicate to the clinician that stricter bowel preparation may be required.

This study also examined the effect of these procedural factors on PDRs with multivariate analysis conducted on the data, demonstrating that longer scope withdrawal times (including intervention times) were associated with a higher detection of polyps (*P* = 0.004). This aligns with the NBCSPQWG who recommends that mean colonoscopy withdrawal time should be 6 min or greater for procedures where no polypectomy is performed to ensure adequate visualization.^24^ This is reaffirmed by recommendations by the European Society for Gastroenterology (ESGE).^18^ The average withdrawal time in our cohort when no polypectomy was performed was 14.02 min, which aligns with these international standards.

This study also examined the relationship between poor bowel cleansing and patients needing to be rebooked within 12 months. The U.S. Multi‐Society task force on CRC recommends that scores of 0 or 1 in any colon segment is associated with a clinically inadequate preparation for mucosal inspection during colonoscopy and therefore warrants repeat examination within 1 year.^19^ Exactly 2.77% of patients received a fair or poor result in the NER1006 group compared with 12.30% of patients in the macrogol and 3 L polyethylene glycol group. The Australian clinical practice guidelines for surveillance colonoscopy have implemented guidelines that advise against repeating a colonoscopy for 10 years and screening no earlier than 5 years after a negative colonoscopy or removal of a low‐risk adenoma/s in asymptomatic patients.^25^ Given that our cohort size was less than 10% of the total colonoscopies conducted at our center each year and specific to NBCSP patients only, the impact of poor bowel cleansing in our cohort was significant as 7.98% of patients required a shorter colonoscopy surveillance interval of 6–12 months due to inadequate bowel preparation.

This study has potential limitations. A source of potential bias was the subjectivity of the individual endoscopists. Although majority of the results were validated through the Boston Bowel Preparation Scale, approximately 20% of results were validated through the AS and then converted to a BBPS equivalent. A BBPS ≥6 was denoted as excellent and good, whereas a BBPS <6 as inadequate, fair, or poor.^11^ However, this is not a universally accepted definition and may vary between clinicians. Furthermore, access to tolerability data was not available and was therefore not examined in this study. This would have allowed us to evaluate the safety, tolerability, and adverse effects of both preparations. This would also allow us to determine whether other methods such as an instructional bowel preparation video could significantly improve patient adherence, demonstrated in a study conducted by Prakash *et al*. in 2013.^26^

In our patient cohort who received the new low‐volume 1 L solution, NER1006 demonstrated high‐quality overall bowel and right‐sided colon cleansing using split‐dose administration. In the NER1006 group, PDRs were higher and there were less patients rebooked due to poor bowel preparation. This is important as this decreases the need for additional washing during colonoscopy; but also decreases the associated costs to the public healthcare system. This is the first study to examine and demonstrate NER1006 superiority compared with a commonly prescribed bowel preparation regime in Australia.

Repeat colonoscopies due to inadequate bowel preparation have significant impacts on both the patient and the public healthcare system. As the incidence of CRCs has increased both in Australia and worldwide, it is imperative that alternate bowel preparation solutions are examined, which can provide adequate cleansing and a favorable peri‐procedural experience for the patient. This retrospective audit has demonstrated that NER1006 is an effective low‐volume preparation option, providing superior cleanliness and a reduced requirement for washing using split‐dose administration regardless of patient characteristics. It is currently the lowest volume PEG‐based solution available on the market and advantageous to patients who do not tolerate high‐volume preparations.
